# Household air pollution and incidence of eclampsia in eight low‐ and middle‐income countries

**DOI:** 10.1002/ijgo.14484

**Published:** 2022-10-12

**Authors:** Tanya Robbins, Katy Kuhrt, Nicola Vousden, Paul Seed, Andrew Shennan

**Affiliations:** ^1^ Department of Women and Children's Health School of Life Course Science, King's College London London UK

**Keywords:** air pollution, eclampsia, pre‐eclampsia, pregnancy

## Abstract

The authors found a strong correlation between national‐level data for deaths from household pollution and eclampsia rates in the CRADLE 3 (Community Blood Pressure Monitoring in Rural Africa & Asia: Detection of Underlying Pre‐Eclampsia and Shock) study clusters (correlation coefficient: 0.84).

Indoor air pollution from solid fuels for cooking and heating predominantly takes place in low‐ and middle‐income countries (LMICs).^1^ Hypertensive disorders in pregnancy (HDPs) exhibit regional variation as a cause of mortality.^2^ A total of 94% of maternal deaths occur in LMIC settings, with 22% attributable to HDPs in urban and peri‐urban areas with simple solutions to prevent them.^2^ Pre‐eclampsia and eclampsia stand out as the major causes of mortality and morbidity among HDPs. Poor pregnancy outcomes are linked to maternal air pollution exposure, although mechanisms remain unclear.^3^ Hypertension is known to enhance the permeability of the blood–brain barrier and impair cerebral autoregulation. Black carbon particles found on the fetal side of the placenta make translocation via the maternal circulation plausible.^4^

We correlated national‐level data for deaths caused by indoor air pollution with eclampsia rates in 10 LMIC sites across eight countries. The authors evaluated eclampsia rates prospectively determined from 2692 eclamptic cases in 536 223 deliveries obtained from our CRADLE 3 (Community Blood Pressure Monitoring in Rural Africa & Asia: Detection of Underlying Pre‐Eclampsia and Shock) trial,^3^ a pragmatic stepped‐wedge randomized controlled trial assessing the impact of a vital signs alert device on a composite outcome of maternal death, emergency hysterectomy, and eclampsia (King's College London Ethics Committee approval: LRS‐14/15–1484).^4^ These countries included urban populations from Ethiopia, Haiti, India, Malawi, Sierra Leone, Uganda (two sites), Zambia (two sites) and Zimbabwe. For the purposes of this analysis all eclamptic fits were included. The total eclampsia rate was 50.2 of 10 000 deliveries (range, 19.6–107.1). Death rates attributable to indoor air pollution were obtained from the Global Burden of Disease Results Tool and the Institute for Health Metrics and Evaluation (https://ourworldindata.org/indoor‐air‐pollution#death‐rates‐from‐indoor‐air‐pollution).

There was a significantly positive correlation between national‐level data for deaths attributable to indoor household pollution and eclampsia rates, with a correlation coefficient of 0.84 (95% CI, 0.468–0.963; *P* = 0.0019). This increased to 0.86 (95% CI, 0.51–0.9; *P* = 0.0013) when eclampsia occurred at home (44.9% of cases). This supports an effect occurring at home, such as indoor air pollution.

Our data suggest that indoor household pollution may account for an acute lowering of seizure threshold in pre‐eclampsia, possibly related to hypoxia. Household air pollution using indoor cooking stoves in Nigeria has been linked to histological measures of chronic placental hypoxia,^5^ and brain cells are sensitive to oxygen deprivation, which can cause seizures.^6^ As these are two independently derived data sets it is difficult to prove causality. We cannot rule out an association attributable to other causes, but this provides a hypothesis for further testing. We were unable to obtain specific data on household pollution and have assumed that the reported deaths from this will include pregnant women at home. Further research is needed to understand the effects and mechanisms to ensure measures are taken to protect those most at risk. This evidence demonstrates that air pollution may impact vulnerable populations the most and may explain some of the observed inequalities in maternal health. Future research should determine whether indoor air pollution increases the prevalence of pre‐eclampsia or increases morbidity from serious manifestations such as eclampsia.

## AUTHOR CONTRIBUTIONS

T.R. was involved in the design, planning, conduct, data analysis, and manuscript writing; K.K. was involved in the data analysis and manuscript writing; N.V. was involved in the design, planning, conduct, and manuscript writing; P.S. was involved in the design, planning, and data analysis; and A.S. was involved in the design, planning, conduct, data analysis, and manuscript writing.

## CONFLICT OF INTEREST

There are no relationships for any author that could compromise the objectivity of the paper and its review.

## Data Availability

Data sharing is not applicable to this article as no new data were created or analyzed in this study.
